# Glucosylceramide acyl chain length is sensed by the glycolipid transfer protein

**DOI:** 10.1371/journal.pone.0209230

**Published:** 2018-12-14

**Authors:** Anders P. E. Backman, Josefin Halin, Henrik Nurmi, Anna Möuts, Matti A. Kjellberg, Peter Mattjus

**Affiliations:** Biochemistry, Faculty of Science and Engineering Åbo Akademi University, Artillerigatan 6A, III, FI Turku, Finland; Virginia Commonwealth University Medical Center, UNITED STATES

## Abstract

The glycolipid transfer protein, GLTP, can be found in the cytoplasm, and it has a FFAT-like motif (two phenylalanines in an acidic tract) that targets it to the endoplasmic reticulum (ER). We have previously shown that GLTP can bind to a transmembrane ER protein, vesicle-associated membrane protein-associated protein A (VAP-A), which is involved in a wide range of ER functions. We have addressed the mechanisms that might regulate the association between GLTP and the VAP proteins by studying the capacity of GLTP to recognize different N-linked acyl chain species of glucosylceramide. We used surface plasmon resonance and a lipid transfer competition assay to show that GLTP prefers shorter N-linked fully saturated acyl chain glucosylceramides, such as C8, C12, and C16, whereas long C18, C20, and C24-glucosylceramides are all bound more weakly and transported more slowly than their shorter counterparts. Changes in the intrinsic GLTP tryptophan fluorescence blueshifts, also indicate a break-point between C16- and C18-glucosylceramide in the GLTP sensing ability. It has long been postulated that GLTP would be a sensor in the sphingolipid synthesis machinery, but how this mechanistically occurs has not been addressed before. It is unclear what proteins the GLTP VAP association would influence. Here we found that if GLTP has a bound GlcCer the association with VAP-A is weaker. We have also used a formula for identifying putative FFAT-domains, and we identified several potential VAP-interactors within the ceramide and sphingolipid synthesis pathways that could be candidates for regulation by GLTP.

## Introduction

Glycosphingolipids are ubiquitous components of eukaryotic membranes and participate in several biological processes, including cell development and proliferation, cancer cell adhesion, and neurodegenerative diseases. The synthesis of sphingolipids occurs via multi-step reactions, and subsequent sphingolipid transport from the site of origin in the endoplasmic reticulum (ER) to their different destinations requires elegant mechanisms. The transport of ceramide, as well as more complex glycosphingolipids, occurs via lipid transfer proteins [1] and by intricate vesicular trafficking machinery [2]. In the cis-Golgi compartment, on the cytosolic leaflet, ceramide is glycosylated to glucosylceramide (GlcCer) by GlcCer synthase [3, 4]. From there, GlcCer is further glycosylated inside the Golgi apparatus, where more complex glycosphingolipids, such as gangliosides and globosides, are formed.

Another transport route for GlcCer is mediated by the action of four-phosphate adaptor protein 2 (FAPP2). FAPP2 translocates GlcCer from the cis-Golgi to the trans-Golgi, where GlcCer is converted to lactosylceramide and globosides [5–7]. FAPP2 consists of an N-terminal pleckstrin homology domain that recognizes the Golgi marker phosphatidylinositol 4-phosphate, a central proline-rich region, and a glycolipid transfer protein (GLTP)–like domain in the C terminus. The involvement of different species of GlcCer in this branching of GlcCers into different classes of higher glycosphingolipids is not clear, especially with respect to the ceramide backbone lengths that are recognized by FAPP2 or the other sensors that may be involved. However, GLTP has been suggested to function as this type of sensor [8, 9]. Therefore, the aim of the present study was to examine how GLTP recognizes GlcCers with different fully saturated acyl chain lengths, ranging from C8 to C24 with respect to their N-linked fatty acids. This was of interest because the early steps in sphingolipid synthesis show a distinct specificity for the substrate chain lengths [10]. The specific ceramides are synthesized by the six ceramide synthases that produce ceramides differing in their acyl chain compositions [10]. These ceramide backbones are then further metabolized into glycosphingolipids and sphingomyelins.

The glycolipid transfer protein GLTP shows a conformational uniqueness and is highly conserved evolutionarily [11, 12]. It folds into a double-layered eight alpha helical structure (GLTP-like domain) that is found in several other proteins [9, 12]. The cytosolic GLTP has a broad specificity towards both simple and more complex glycosphingolipids [11]. However, among all glycosphingolipids, only GlcCer appears to be oriented toward the cytosol in the Golgi apparatus [1]. Therefore, GlcCer is the most likely glycosphingolipid that would be accessible to GLTP.

The enzymes involved in the synthesis of complex glycosphingolipids, such as gangliosides and globosides, are all lumenally oriented in the Golgi and trans-Golgi network [1]. The complex glycosphingolipids therefore end up on the outer leaflet of the plasma membranes in most cells, following their synthesis and sorting events. The endocytosed plasma membrane glycosphingolipids, on the other hand, are destined for the lumen of the lysosomes or for recycling back to the plasma membrane [13]. Some studies have also reported the presence of gangliosides (GM_1_ and GD_1_) in the nuclear envelope, where they are tightly associated with sodium and calcium, and in the endonuclear compartment [14–17], as well as in conjunction with mitochondrial-associated ER membranes [18–20]. Therefore, the possibility exists that GLTP could also be involved in sensing other more complex glycosphingolipids.

The vesicle-associated membrane protein (VAMP)**-**associated proteins (VAPs) have emerged as platforms for docking various FFAT proteins and FFAT-like proteins (which possess two phenylalanine residues in an acidic tract) at the ER membrane. The VAP proteins are important in bringing together lipid binding proteins [21]. VAPs form homo- and heterodimers, and can interact with protein partners carrying a FFAT motif [22–24].

Here, we exploit the high sensitivity and suitability of a surface plasmon resonance (SPR) method for studying temporary and weak molecular interactions to measure the specificity of GLTP for GlcCers with N-linked acyl chains of varying lengths. We also use tryptophan emission fluorescence to measure the binding of the different GlcCers by GLTP, as well as lipid transfer assays and a thermal shift assay. We found a significantly stronger binding and transfer of GLTP to shorter C8:0-, C12:0-, and C16:0-GlcCers than to longer C18:0-, C20:0-, and C24:0-GlcCers. We also used an algorithm that identifies VAP-binding domains in proteins related to sphingolipid synthesis to explain the possible biological function underlying the ability of GLTP to sense GlcCers of different compositions.

## Materials and methods

### Materials

All phospho- and glycosphingolipids were from Avanti Polar Lipids (Alabaster, AL] except for C20-GlcCer and C24-GlcCer, which were synthesized according to the procedure described below. TPA was synthesized in house from the methyl ester of α-linolenic acid [25]. CPM was obtained from ThermoFisher Scientific.

### Glucosylceramide synthesis

Arachidonic acid (C20:0) and lignoceric acid (24:0) were obtained from Sigma Chemicals (St. Louis, MO) and lyso-GlcCer (d-glucosyl-β1–1′-d-erythro-sphingosine) from Avanti Polar Lipids. GlcCers were synthesized from the lyso-derivatives and fatty acids according to a modified version of the method for sphingomyelin synthesis [26]. The lyso-glucosylceramide and the fatty acid (lyso-sphingolipid/fatty acid, 1:10 molar ratio) were dissolved in dichloromethane/methanol (11:1, v/v) containing 1 vol% trimethylamine. N,N′-dicyclohexylcarbodiimide (DCC; Fluka, Buchs, Switzerland) was added as a catalyst (DCC/fatty acid, 1.2:1, molar ratio) and the reactions were carried out at 35°C for 3 h. The GlcCers were purified by reverse-phase HPLC on a preparative Supelco Discovery C18 column (250 × 21.2 mm, 5 μm particle size) with methanol as the eluent. The purity and identity of the products were verified with HPTLC and the molecular ions verified by MS analysis.

### Protein expression and purification

Bovine GLTP was produced using a pQE-9-GLTP expression vector in *E*. *coli* and separated using its 6× histidine tag. The combined pure GLTP protein fractions were dialyzed twice overnight against a dialysis/SPR running buffer, pH 7.4, consisting of 50 mM Tris, 150 mM NaCl, and 1 mM dithiothreitol. The GLTP for use with the SPR approach needs to be of high purity. Small differences in the buffer conditions result in large variations in the SPR response. Therefore, using the same buffer throughout all experimental steps is important, as well as the final steps in the protein purification procedure [27]. The protein solution was filtered (0.2 μm) and stored at 4°C for a maximum of 7 days. The protein concentration was determined according to the Lowry method [28]. The purity was judged by SDS-PAGE with Coomassie or silver staining. The protein activity was determined as described below.

### Preparation of phospholipid vesicles

Lipid stock solutions in hexane:2-propanol (3:2, v/v) were mixed in the desired amounts, dried under nitrogen, and redissolved in 140 mM NaCl, 20 mM NaH_2_PO_4_, 1 mM DTT, and 1 mM EDTA (pH 7.4). For the lipid transfer assay, the suspension was vortexed and sonicated for 5 min using a Branson 250 titanium probe sonicator (micro tip with a diameter of 3 mm) and then centrifuged for 15 min at 15000 g to remove multilamellar aggregates and titanium probe particles. The POPC acceptor vesicles used in the transfer assay were prepared by sonication. Vesicles for the SPR measurements were made from the dried lipid stock solutions, which were redissolved in 140 mM NaCl, 20 mM NaH_2_PO_4_, 1 mM DTT, and 1 mM EDTA pH 7.4 and kept at 60°C to hydrate for at least 30 min prior to filtration. The lipid solution was extruded 15 times through a 100 nm filter using the Avanti mini extruder (Avanti Polar Lipids).

### CPM thermal shift assay

CPM was used to determine the thermal denaturation of GLTP in the presence of GlcCer [29, 30]. GLTP (0.42 μM) in PBS was incubated with GlcCer (4.2 μM in DMSO) for 30 min at the starting temperature. CPM dissolved in DMSO was added to a final concentration of 6.1 μM, thoroughly mixed in the cuvette, and heated with a ramp rate of 2°C/min (Varian Cary fluorometer). The excitation wavelength was 387 nm, and the emission wavelength was 463 nm. Assays were performed in triplicate during 40 min over a temperature range starting at 20°C and ending at 90°C.

### Tryptophan emission

The interaction between GLTP and glucosylceramide was studied by monitoring the changes in the tryptophan fluorescence emission spectra [31–33]. To a buffer (3 ml PBS with 1 mM DTT) containing 60 μg (0.84 μM) GLTP, increasing amount of lipids dissolved in ethanol were added (1.275 mM per addition). Tryptophan fluorescence (295 nm excitation) was measured between 310 and 420 nm at 37°C on a Cary Eclipse spectrofluorometer with excitation bandwidth at 5 nm and emission bandwidth at 5 nm. The final ethanol concentration was less than 0.2%.

### Surface plasmon resonance

SPR is an optical method for measuring changes in the refractive index upon increase or decrease in mass on the sensor surface. The sensor chip for capturing lipid vesicles is a functionalized dextran gel that is attached to the gold surface of the sensor via a self-assembled monolayer of hydroxyalkyl thiols [34]. The setup of the experiments for determining transfer activity of GLTP was based on the protocol used by Ohvo-Rekilä and Mattjus [35], using a SPR-Navi 200 instrument (BioNavis Tampere, Finland). Before vesicles were loaded, the sensor chip was washed twice with CHAPS detergent to establish a stable base line. Vesicles were bound to the sensor by injecting a 0.5 mM concentration of lipids for 10 min. Unbound vesicles were removed by washing the sensor with a 50 mM solution of NaOH in buffer. GLTP was injected (0.1 μg/μl) and applied for 10 min at a flow rate of 10 μl/min at 23°C.

### Transfer assay

The donor vesicles were composed of 1 mol% TopFluor-GlcCer, 1 mol% competitor lipid, 3 mol% of the nontransferable fluorescence quencher DiI-C18, and 95 mol% POPC or DOPC. A quartz cuvette containing sodium phosphate buffer (total assay 3 ml) was allowed to equilibrate at 37°C under stirring. Donor vesicles (40 nmol lipids) in 100 μl sodium phosphate buffer was added to the assay buffer and were allowed to equilibrate for another 3 min. Acceptor vesicles in 40 μl (400 nmol final lipid concentration) were then added and allowed to equilibrate. The transfer assay was started by addition of 0.25–2 μg of GLTP. The transfer rates during the first minute after GLTP injection, termed the initial transfer rate, can be calculated by determining the increase in the fluorescence intensity (BODIPY-GlcCer unquenching) and comparing this value to the total fluorescence intensity obtained after adding Triton X-100 (final concentration 1%) and subtracting a Triton X-100 blank [27]. Each experiment (donor vesicle preparation) was repeated at least three times, and at least three transfer measurements were conducted per experiment. The data points are averages of all the transfer rates measurements.

### Competition assay

Unlabeled GlcCer (1 mol%) was added to the donor vesicles composed of either POPC or DOPC, as described above. The addition of an unlabeled glycosphingolipid would result in a decrease in the transfer rate of the fluorescently labeled lipid, if the added GlcCer was competing with the TopFluor-GlcCer [27, 36]. If the added lipids are not substrates for the transfer protein, no deviation in the slope of the transfer rate for TopFluor-GlcCer would occur. With this assay, we were able to indirectly determine if the transfer rate of different GlcCers depended on the acyl chain length.

### Lifetime analysis of trans-parinaric acid

The ability of tPA to partition into ordered domains was used to determine the formation of ordered phases in POPC with various lengths of GlcCers (10 mol%). The excited state lifetime of tPA is stabilized in gel phases, in part because of less diffusion-induced quenching by water and oxygen [37]. The samples contained 1 mol% tPA and measurements were performed at a constant temperature (23°C) with a FluoTime 100 instrument (PicoQuant GmbH, Berlin, Germany) using a PLS LED laser source for excitation (298 nm). Emission was recorded above 350 nm. FluoFit Pro software obtained from PicoQuant was used to analyze lifetimes.

### Protein structure analysis

We obtained the crystal structure information of GLTP from the RCSB Protein Data Bank (www.rcsb.org). The GLTP structures were aligned using the mammalian GLTPs, together with the structural conformation of the structure bound lipids using PyMOL (www.pymol.org).

### VAP binding domain analysis

We used Levine’s algorithm to identify putative FFAT-domains [38]. The analyzed proteins were known to associate with the precursors of glycosphingolipid synthesis, as well as with proteins involved in the sphingomyelin pathway. The FFAT “two phenylalanines in an acidic tract” motif is present in many VAP binding proteins; however, a much broader VAPome can be identified using an algorithm that identifies FFAT-like motifs and classifies potential VAP-binding proteins according to a ranking. The canonical FFAT motifs score 0, and divergences score a higher number, the “Levine factor,” thereby predicting probable FFAT-domains and ranks the by similarity to the canonical FFAT-domain sequence.

### GLTP/VAP-A pull-down in presence of glycolipids and Western blotting analysis

Bovine His-GLTP was produced as described in [34]. Human and N-terminally GST-tagged VAP-A was produced as described in [34], where the His-beads had been exchanged for Gluthatione Sepharose 4 Fast Flow beads (GE Healthcare) and the final purification followed the protocol described by GE Healthcare. To analyze whether GLTPs binding to VAP-A would differ, while having bound different acyl-chain length glucocylceramides, we mixed 0.5 μM His-bGLTP with 5 μM glycolipid, dissolved in DMSO, in 1xPBS, pH 7.4, for 45 min at 37°C. 2,5 μM GST-VAPA was then added to the samples, which were further incubated for 30 min at 37°C. Glutathione Sepharose 4 Fast Flow beads, in a slurry of 1:1 beads/PBS 0.1% BSA was added together with 500 ul of wash buffer (20 mM Tris-HCl, 150 mM NaCl, 1 mM EDTA, 10% glycerol, 0.1% Triton X-100, pH 7.4) and was mixed for 1 h at 4°C. The beads were collected by centrifugation and washed three times with the wash buffer. The samples were then denatured at 95°C for 10 min in Laemmli buffer. Western blotting was used for analysis of the samples.

## Results

The objective of this work was to determine the binding specificity of GLTP for saturated GlcCers with different N-linked acyl chain lengths. Several previous crystallographic studies reported the crystallization of GLTP with different acyl chain length glycosphingolipids (for review see, [12]). However, the binding of glycolipids by GLTP under crystallization conditions is not readily comparable to binding in a fully hydrated environment, such as that occurring during GLTP glycolipid binding and transfer from bilayer membranes. The affinity of GLTP for GlcCers of different lengths also has not previously been addressed systematically. Since GLTP is a cytosolic protein [39], and GlcCer, unlike the other mammalian glycosphingolipids, has a cytosolic orientation on the outer leaflet of the early Golgi [40, 41], this affinity is of particular biological interest. One often used glycosphingolipid in GLTP studies is galactosylceramide; however, it is localized inside the ER [42, 43].

### Changes in GLTP melting temperature is caused by GlcCer binding

**Fig 1:**
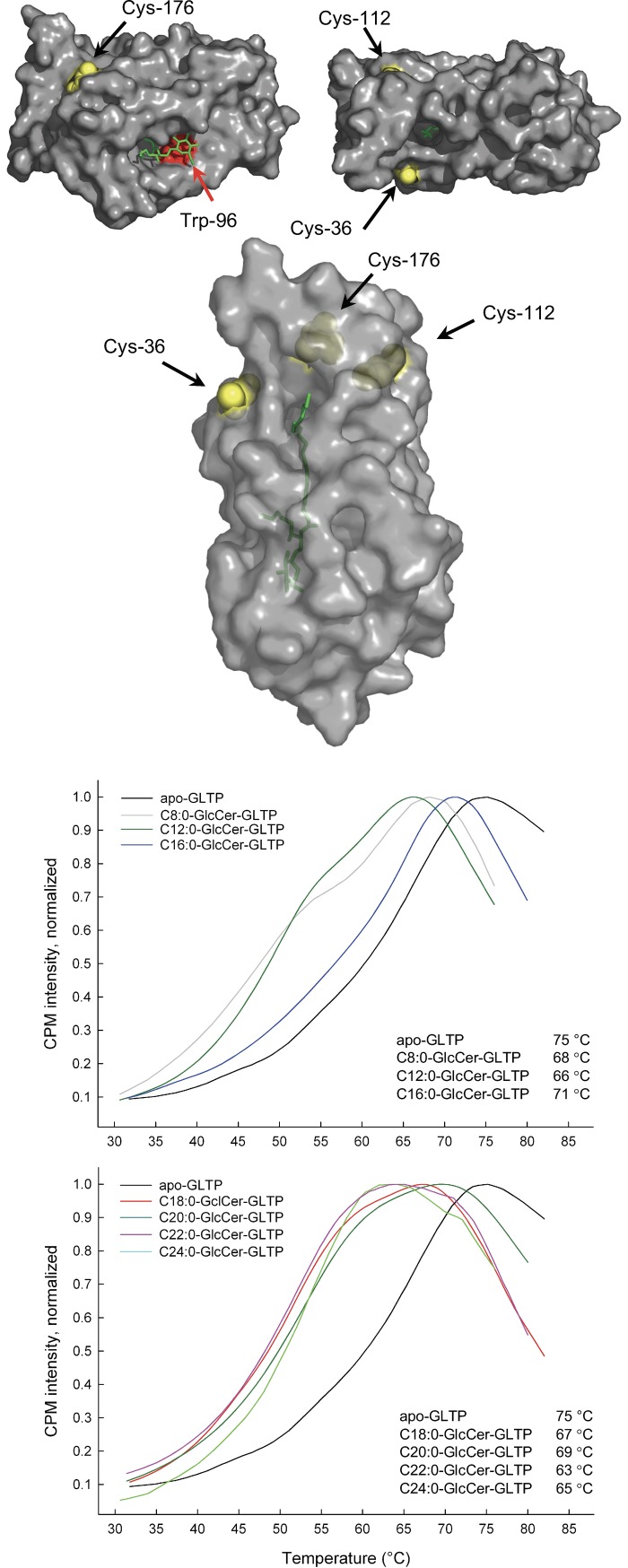
GLTP structure and CPM thermal shift assays. The crystal structure of GLTP with the three cysteine (Cys) residues in yellow, bound ganglioside GM_3_ (2BV7) in green, and the tryptophan Trp-96 in red. The left structure shows the opening of the hydrophobic tunnel, with the N-linked acyl chain and saccharide headgroup of GM_3_ visible. On the right, GLTP is turned horizontally 180 degrees, exposing the opening of the tunnel end. The third structure show the exposed Cys-36 on the surface of GLTP and the two more embedded Cys-112 and Cys-176. The lower graphs show the increase in the CPM fluorescence intensity (λ_exc._ 400 nm) at 500 nm as a function of a temperature increase. The black trace corresponds to wildtype apo-GLTP (0.42 μM) in PBS buffer. The different colored traces are GLTP incubated with GlcCer of different lengths (ethanol injected) 30 min prior to heating. The curves are averages of at least 4 independent measurements. The curves were normalized to the intensity maximum for the apo-GLTP melting curve. The curve maximas are shown for each representative GlcCer-GLTP mixture.

We initially examined the changes in protein stability occurring in the presence of different GlcCers. GLTP contains three cysteine (Cys) residues, at positions 36, 112 and 176 (Fig 1). Cys-112 and Cys-176 are 9.5 Å apart from each other, as measured from the C_α_-atoms in the 2BV7 structure with lactosylceramide bound [44]. However, a movement of α-helix 8 might bring Cys-176 sufficiently close to Cys-112 located in α-helix 4 to allow intramolecular disulfide bridging [9, 44]. This movement could represent a potential mechanism for regulating the transfer activity of GLTP.

Cys-36 in α-helix 1 is located on the surface of GLTP (Fig 1). Abe and Sasaki [45] suggested that Cys-36 could form a disulfide bond with the respective cysteine in another GLTP and form a dimer. This dimer has almost no transfer activity and is probably not a biologically active form [44]. The hydrophobic tunnel that accommodates the glycosphingolipid stretches close to the Cys-36 residue (Fig 1). However, the proximity of Cys-36 to the back end of the hydrophobic cavity of GLTP prompted us to analyze the melting temperature of GLTP, using CPM (N-[4-(7-diethylamino-4-methyl-3-coumarinyl)phenyl]maleimide) as a function of GlcCer. CPM reacts specifically with the side chain of free cysteines and forms a fluorescent complex. The front opening of the cavity contains a tryptophan (Trp-96), which forms the bottom of the surface depression involved in the saccharide head group recognition site (Fig 1).

The melting temperature of 75.0°C for wildtype GLTP (Fig 1; black trace) is higher than for GLTP with bound GlcCers. We interpret the lower melting temperature of GLTP with bound GlcCers as a better solvent accessibility to the cysteine-CPM complex, compared to the apo-GLTP form. The empty, collapsed hydrophobic tunnel of the apo-GLTP allows for a low solvent accessibility and consequently has the highest melting temperature. CPM is not sensitive enough to significantly report differences in the melting temperature as a function of GlcCer acyl chain lengths. However, this initial finding prompted us to further analyze how different GlcCers are sensed by GLTP.

### Chain specific GlcCer transfer from POPC vesicles measured with SPR

**Fig 2:**
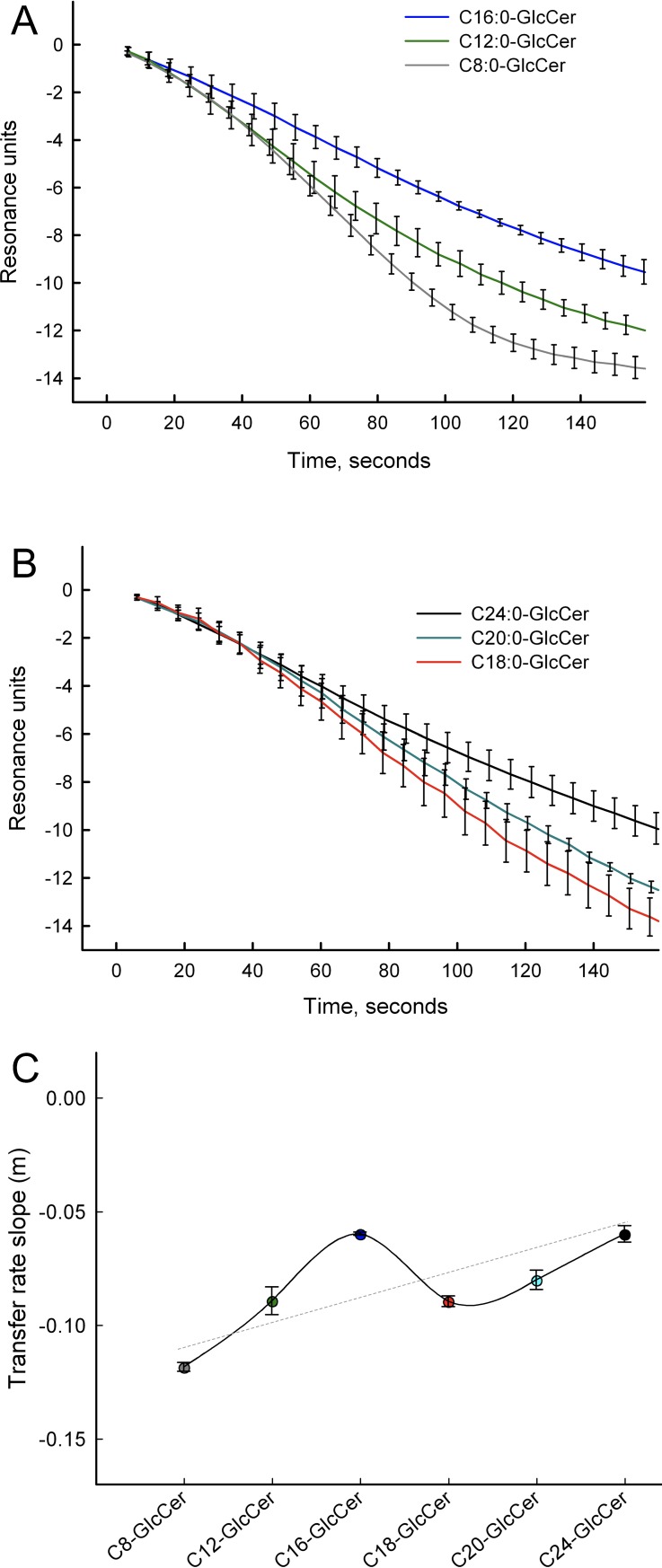
GLTP mediated transfer of GlcCer measured by SPR. POPC vesicles containing 10 mol% GlcCers with various N-linked acyl chain lengths were adsorbed to the SPR chip. (**A**) Short GlcCer (C8:0-, C12:0-, and C16:0-GlcCer) transfer (0.1 mg/ml GLTP, flow rate of 10 μl/min) from vesicles is seen as a loss in resonance units as a function of time. **(B)** Long GlcCers, (C18:0, C20:0, and C24:0) transfer from POPC vesicles is mediated by GLTP. (**C**) The line shows the slope as a function of the increasing acyl chain lengths of the GlcCers. The SPR curves and values for the slopes represent an average of at least four different SPR runs. The linear regression line is shown in gray.

We examined the ability of GLTP to transfer GlcCer with different N-linked acyl chains using the previously developed GLTP SPR assay [35]. Immobilized vesicles consisting of 1-palmitoyl 2-oleoyl phosphatidylcholine (POPC) and 10 mol% GlcCer were bound to a gold sensor, as described in the Materials and Methods [35]. Addition of GLTP (0.1 mg/ml) in a Tris-HCl buffer lowers the SPR response as a function of time, due to a loss of mass because of GLTP binding and removal of GlcCer from the immobilized vesicles (S1 Fig). No loss of material from the chip occurs before GLTP is added. The fastest rates were seen for the short GlcCers (Fig 2A), whereas a slower GLTP-mediated transfer was seen for longer GlcCers (Fig 2B).

We also conducted control experiments using immobilized vesicles containing only POPC or POPC/C18:0-GlcCer and both wild type GLTP and a transfer-inactive mutant GLTP, W96A. We obtained only small changes in the SPR resonance units for W96A GLTP when added to POPC/C18:0-GlcCer vesicles (S2 Fig). However, wildtype GLTP removed C18:0-GlcCer from POPC/C18:0-GlcCer (90:10, mol%/mol%) immobilized vesicles (S2 Fig, red line).

The line in Fig 2C represents the slope in the transfer curve for the different GlcCers transported by GLTP. The transfer rates are fastest for the short GlcCers and slowest for the longer GlcCers. The transfer rate slopes changed almost linearly as a function of increasing N-linked acyl chain length of the GlcCers, with a deviation for the C16-GlcCer transfer rate. We do not yet have an explanation for this deviation of C16:0-GlcCer from the other analyzed GlcCers. However, we speculate that the POPC membrane matrix could allow for good acyl chain matching and intermolecular interaction between C16-GlcCer and the matrix lipids, compared to the other GlcCer lengths that interact weaker with POPC but favorably with themselves. The initial transfer rate of GlcCer by GLTP was calculated from the SPR data for the first 100 seconds (S3A Fig). The SPR curves from at least 5 independent experiments for each GlcCer were combined and averaged, and the slopes were calculated using linear regression, as illustrated in S3B Fig.

### Competition transfer assay

**Fig 3:**
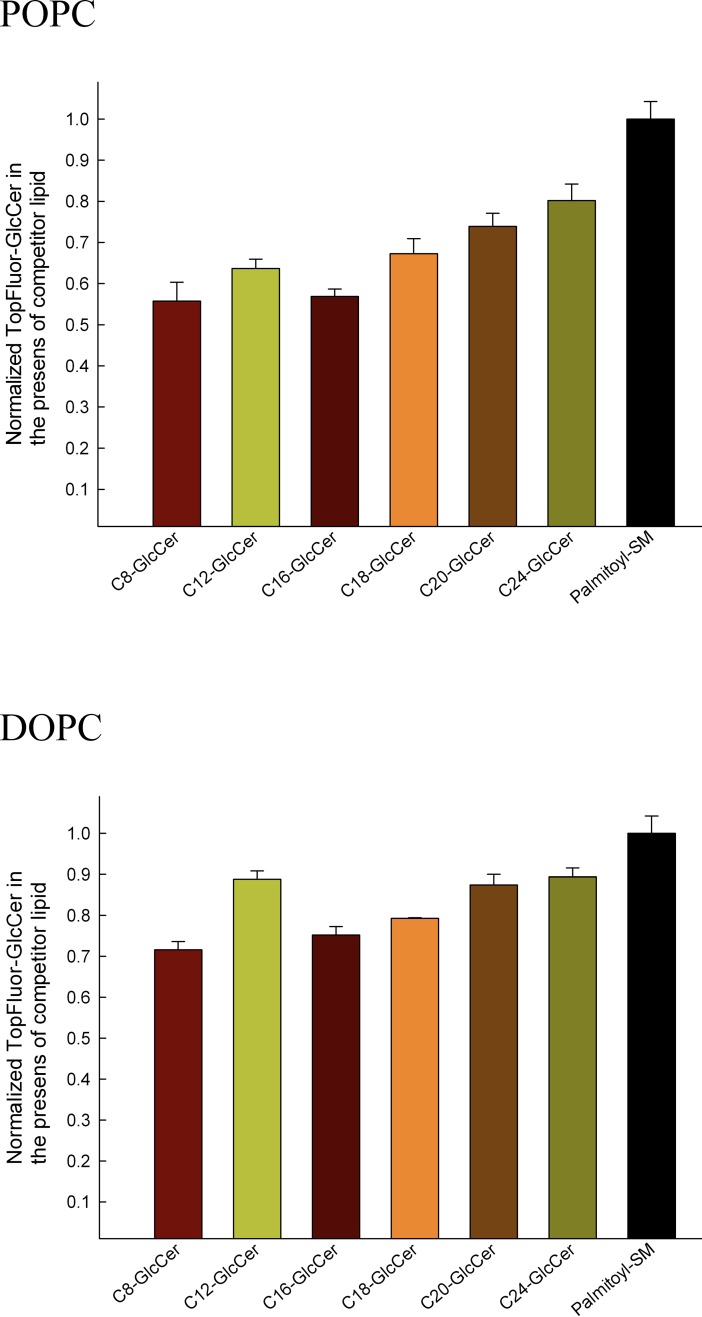
Competition transfer assay measured GlcCer transfer rates. Unlabeled GlcCers (1 mol%) in POPC or DOPC compete with TopFluor-GlcCer as substrates for GLTP. The rate for TopFluor-GlcCer transfer in the non-competing PSM lipid sample was normalized to 1. The data are from at least three different experiments. A statistically significant difference occurred between all fully saturated GlcCer competitor lipids and the PSM control, black bars (P = 0.001).

We further examined the ability of GLTP to recognize and transfer GlcCer of different lengths using an indirect method previously developed for analyzing the substrate specificity of lipid transfer proteins [27, 36]. Since GLTP does not transfer sphingomyelin, the transfer of TopFluor-GlcCer by GLTP was unaffected by the addition of palmitoyl-sphingomyelin (PSM) as a competitor. This transfer rate was used as the control rate, and it represented the maximum transfer rate (Fig 3, black bars). A reduction in transfer rate of TopFluor-GlcCer indicated a greater competition by the unlabeled lipid and identified it as a better substrate for GLTP. We used this competition transfer assay to measure the GLTP-mediated transfer of TopFluor-GlcCer in the presence of an unlabeled GlcCer (Fig 3). The transfer of the fluorescent GlcCer was significantly lowered when short GlcCers were present in the same donor vesicle population, but the competition for longer GlcCers was not as strong. This result was seen for vesicles composed of either POPC or dioleoylphosphatidylcholine (DOPC) containing 1 mol% of the competitor lipid GlcCer.

### Lifetime measurements

The GLTP-mediated transfer of glycosphingolipids is complex and is influenced by the glycosphingolipid carbohydrate head group and the acyl chain structure of the ceramide backbone, as well as the physical and chemical properties of the host membrane that harbors the glycosphingolipid. The mixing properties of the glycosphingolipids in the host membrane is an important determinant for the ability of GLTP to find, extract, and remove the lipid from the membrane [46–48]. A membrane that is tightly packed and contains sphingomyelin will show a very slow transfer of glycosphingolipids, whereas loosely packed membranes, such as those consisting of POPC and DOPC, will show rapid transfer [9, 49, 50].

The GLTP-mediated transfer seen in our experiments, as well as in biological membranes, is likely to depend, in part, on how the different chain length-GlcCers are mixed within the host membrane. Therefore, we examined the membrane packing properties using 10 mol% GlcCer in POPC vesicles and a trans-parinaric acid (TPA) probe, which shows sensitivity to membrane packing. The TPA lifetime is short in fluid membranes (<5 ns), intermediate in a liquid-ordered phase, and long (30 ns and longer) in gel and solid membranes [37, 51, 52]. TPA shows a preference for partitioning into ordered/gel-phase domains.

**Fig 4:**
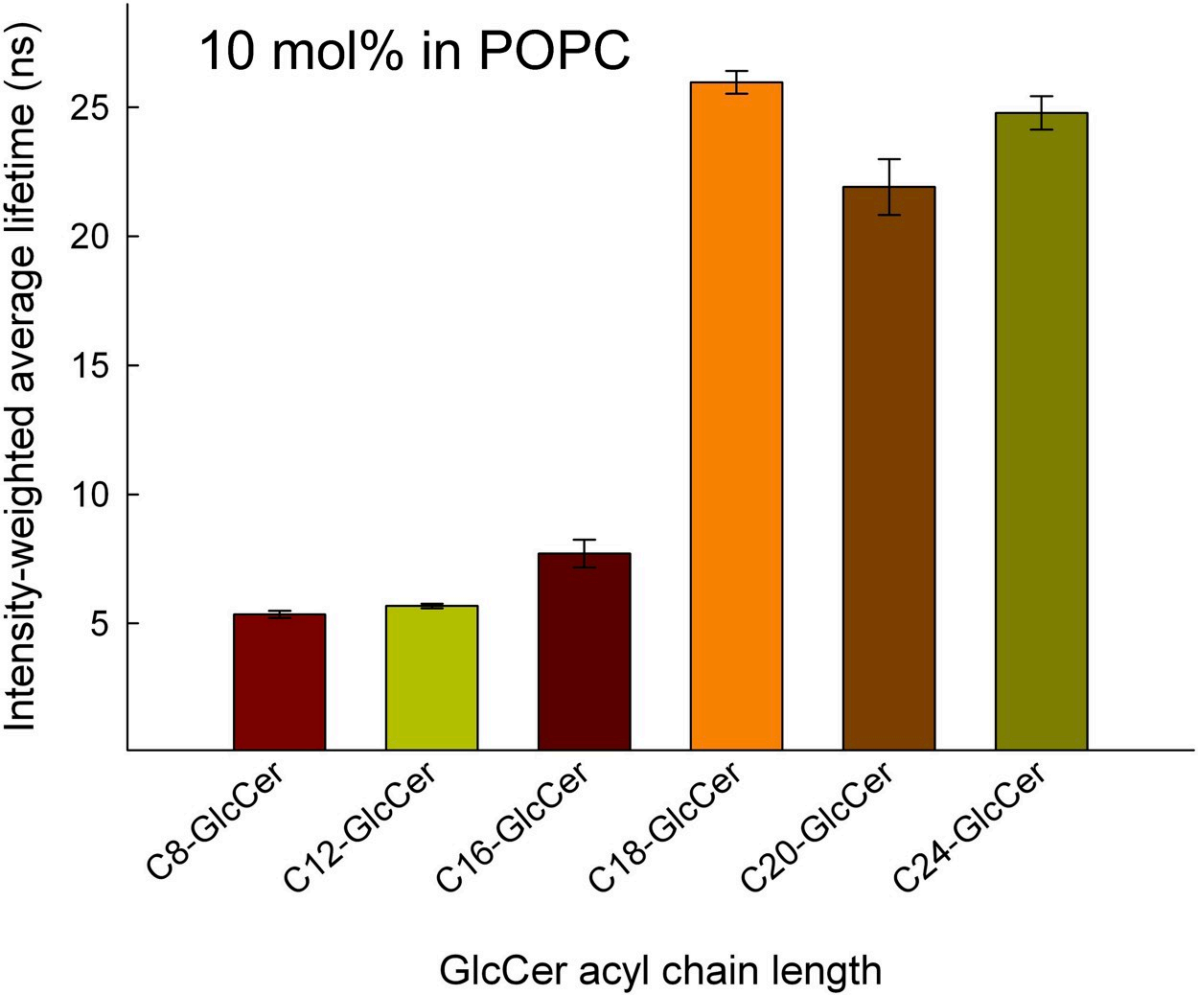
Intensity-weighted average lifetime analysis as a function of bilayer vesicle composition. POPC bilayer vesicles (50 μM) contained 10 mol% fully saturated GlcCer of different N-linked acyl chain length and 1 mol % trans-parinaric acid. The trans-parinaric acid (TPA) lifetime is short in fluid membranes (<5 ns), intermediate in a liquid-ordered phases, and long (35 ns) in gel and solid membranes. Average lifetimes (ns) are shown for each composition, with average ± SD for at least three independent measurements.

We used the TPA lifetime analysis to detect the possible formation of ordered phases in POPC when different acyl chain lengths of GlcCer were added (Fig 4). The presence of shorter GlcCers in the POPC membranes (up to C16:0-GlcCer) resulted in a short TPA lifetime (under 10 ns), whereas longer GlcCers (C18:0-GlcCer and longer) extended the TPA lifetime to between 20 and 25 ns. The phase states for the C8:0-, C12:0-, and C16:0-GlcCer at 10 mol% in POPC is likely to be in a fluid phase with very low gel phase formation. For the longer C18:0-, C20:0-, and C24:0-GlcCer, a significantly longer lifetime would indicate that the GlcCer clusters into a gel phase and allows for a better partition of TPA into a more tightly packed environment. This indicates that the differences in the GLTP binding and transfer rates that we show here for the different GlcCers cannot solely be attributed to the GlcCer phase states.

### Tryptophan emission

GLTP is intrinsically fluorescent due to the presence of three Trp and ten Tyr amino acids among its 209 residues. Only one, Trp-96, is directly involved in the glycosphingolipid binding site [12]. The glycosphingolipid binding to GLTP, rather than the membrane association, is mainly responsible for the emission signature change in Trp fluorescence of GLTP [53, 54]. Crystallographic analysis of GLTP reveals that the three Trp residues at positions 85, 96, and 142 are all located close to the glycosphingolipid binding site [12]. Earlier studies showed that glycosphingolipid binding alters the environment of Trp-96 and that Trp-96 accounts for 70–75% of the total emission intensity of the three Trp residues of GLTP [53, 55]. The wild-type GLTP and a GLTP mutant with only the Trp in position 96 remaining showed a clear blueshift in the presence of membranes. No blueshift was seen for mutants with Trp residues remaining at either position 85 or 142 [53]. Furthermore, a Trp-96-phenylalanine GLTP mutant capable of transferring glycosphingolipids showed no shift in the Trp emission signal [54]. Therefore, we used the Trp emission as a measurement of how well the GlcCer affect the emission blueshift.

**Fig 5:**
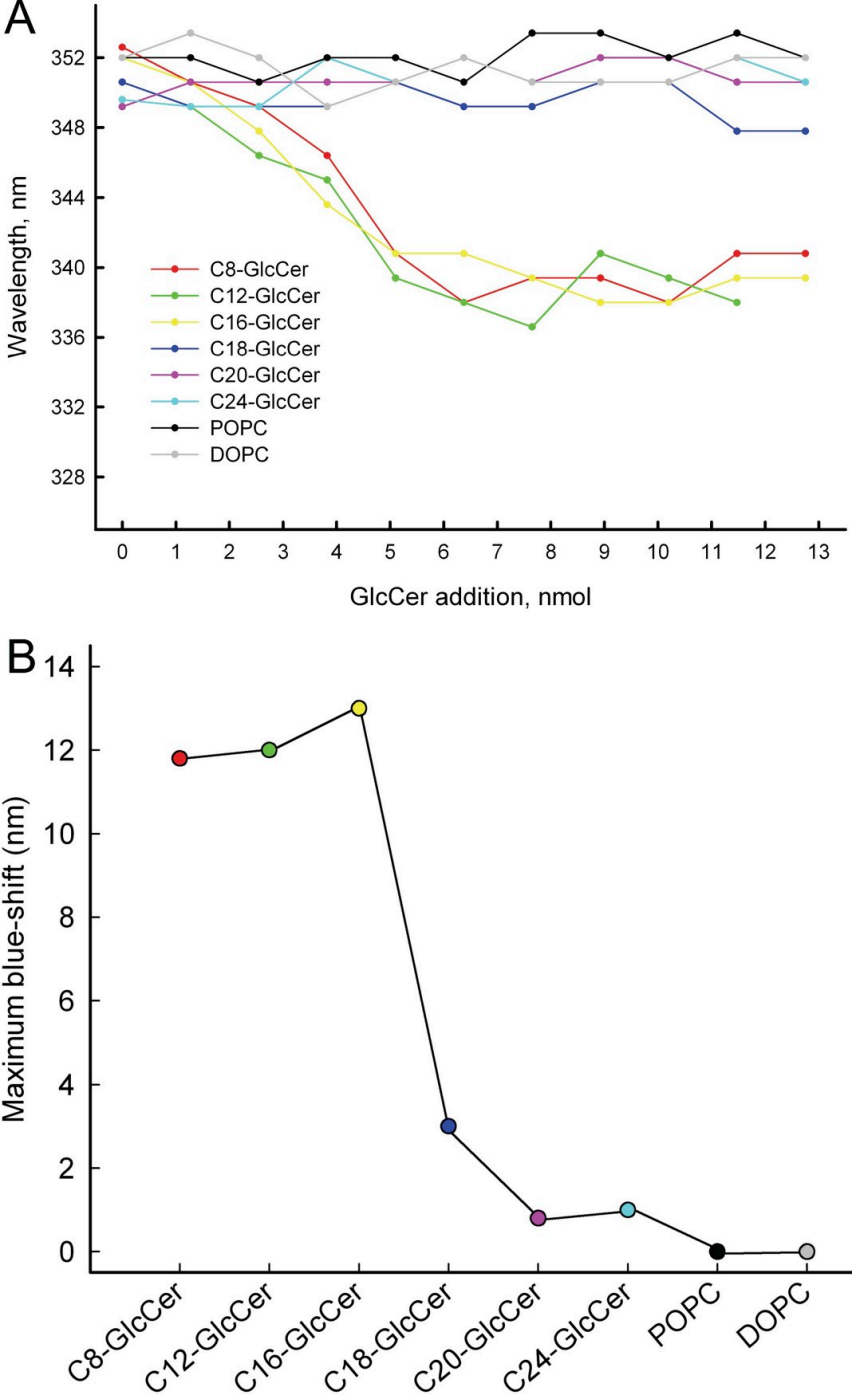
Changes in GLTP Trp emission maxima versus GlcCer concentration. **(A)** Trp emission maxima versus increasing amounts of GlcCer. **(B)** C8:0-GlcCer, C12:0-GlcCer, and C16:0-GlcCer created blueshifts between 12 and 13 nm, whereas the longer GlcCers, as well as POPC and DOPC, showed no significant blueshifts. The data are from at least three different experiments.

Previous studies showed that binding of glycosphingolipids to wildtype GLTP cause different blueshifts in the tryptophan emission [53, 54]. Zhai and coworkers observed a slower GLTP binding equilibration when simple glycolipids (LacCer) contained long saturated acyl chains [54]. Here we determined if the emission shifts differ for uptake of short versus long GlcCers by ethanol-injecting the same amount of lipid (4.2 μM) into a solution of wildtype GLTP (0.42 μM) and recording the emission spectrum from 310 to 420 nm. Addition of GlcCer at increasing concentrations resulted in different emission blueshifts that depended on the length of the GlcCer added (S4 Fig). No emission shift was noted with increasing POPC (S4 Fig) or DOPC (data not shown). The change in the emission maxima as a function of increasing GlcCer concentration is shown in Fig 5A. C8:0-GlcCer, C12:0-GlcCer, and C16:0-GlcCer all show a blueshift between 12 and 13 nm. The longer GlcCers, as well as POPC and DOPC, show no significant shifts. The maximum blueshift for each GlcCer is shown in Fig 5B The C8:0-, C12:0-, and C16:0-GlcCers are able to cause a significantly larger shift in the Trp emission when compared to the longer C18:0-, C20:0-, and C24:0-GlcCer. This means that the uptake of the shorter GlcCers in the binding cavity of GLTP generates a more hydrophobic interior around Trp-96. This finding, together with the SPR and transfer data, indicate that shorter GlcCers are bound and transferred better by GLTP than are the longer variants.

### GLTP and VAP-A pull-down in the presence of glycolipids

**Fig 6:**
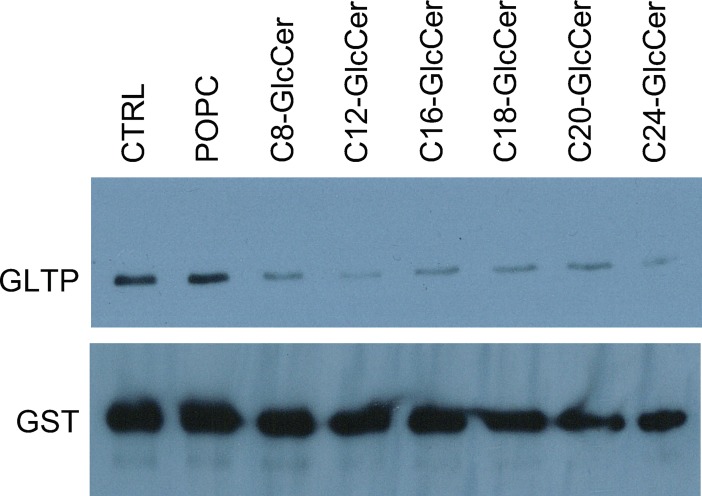
GlcCer dependent GLTP and VAP-A binding. Pull-down assays with subsequent Western blott analysis of GLTP with bound GlcCer and VAP-A. The GST-tagged VAP-A was pulled-down using glutathione sepharose beads and washed, carrying along any bound GLTP. The upper blot is anti-GLTP and the lower blot anti-GST antibodies. In the lane indicated with CTRL only GLTP and VAP-A was pulled down, the lane indicated POPC contains GLTP, POPC and VAP-A. The blot shown is representative of three different independent experiments.

We examined GLTP’s binding to VAP-A in the presence of different N-linked acyl chains of GlcCer, in order to examine if this interaction would change because of the GlcCer bound inside GLTP’s hydrophobic tunnel. GLTP was incubated in the presence of different acyl-chain length glycolipids, before addition of VAP-A, containing a Glutathione Sepharose Tag. The tagged VAP-A was pulled-down using glutathione sepharose beads and washed, carrying along any bound GLTP. The presence and amount of GLTP in the samples was analyzed using Western blot, to analyze the effect GlcCer binding had on GLTP/VAP-A binding. We found that GlcCer affects GLTP’s ability to bind VAP-A (Fig 6). If GLTP has a bound GlcCer the association with VAP-A is weaker. An explanation to this would be the bound glycolipid manipulating the FFAT-domain from within GLTP and effectively “turning off” the GLTP/VAP-A interaction. However, this finding needs to be investigated further.

### How does the bound lipid fit into the hydrophobic cavity of GLTP?

**Fig 7:**
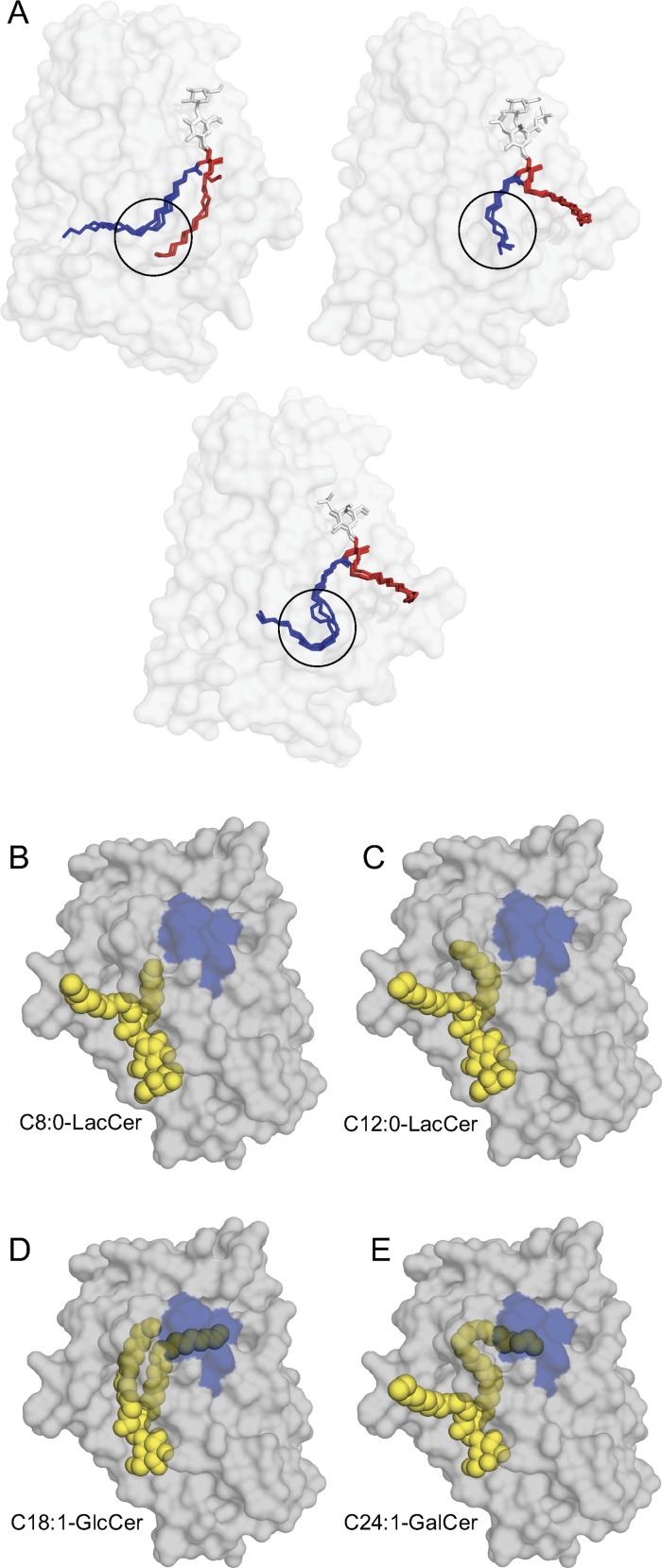
Glycosphingolipid N-linked acyl chain binding in GLTP crystal structures. **(A)** To the left bound C18:1-GlcCer, C18:0-LacCer and ganglioside GM3 lipids in the sphingosine-in conformation found in the crystal structures of different GLTPs (3S0K, 1SX6 and 2BV7 respectively). On the right is bound 12:0-di-sulfatide, C12:0-sulfatide and C12:0-LacCer in the sphingosine-out conformation found in the crystal structures of different GLTPs (4GIX, 4H2Z and 2EVD respectively). The middle lower picture shows bound C24:1-sulfatide, C18:2-GalCer and C24:1-GalCer in the crystallized GLTP structures 3RZN, 2EVL and 2EUK respectively. The carbohydrate head groups are shown in white, sphingosine chains in red, and the N-linked acyl chains in blue. The sphingolipids all fit into the GLTP hydrophobic cavity in a similar manner, with a special kink area (black circle) where the N-linked acyl chains bend. **(B)** GLTP crystal structure (FFAT-like domain in blue) with bound C8:0-LacCer in the sphingosine-out conformation (PDB ID, 2EUM), **(C)** GLTP with bound C12:0-LacCer in the sphingosine-out conformation (PDB ID, 2EVD), **(D)** GLTP with bound C18:1-GlcCer in the sphingosine-in conformation (PDB ID, 3S0K), and **(E)** GLTP with bound C24:1-GalCer in the sphingosine-out conformation (PDB ID, 2EUK).

An analysis of the previously published crystal structure data reveals that the hydrophobic chamber of GLTP that houses the bound lipids is very flexible and appears to conform to the bound lipid [44, 56, 57]. Two different modes of sphingolipid binding have been reported: a sphingosine-in and a sphingosine-out form [12]. In the sphingosine-out binding mode, the sphingoid chain of the ceramide backbone remains outside the hydrophobic pocket. No crystal structures have been reported where the N-linked acyl chain would not be inside the hydrophobic tunnel. Regardless of whether the bound lipid is in the sphingosine-in or sphingosine-out conformation, or contains a saturated or unsaturated lipid chain a kink is observed in the bound N-linked lipid-chain just before the FFAT-domain of the GLTP, at amino acid positions 32 to 38 (Fig 7A). This means that a long N-linked acyl chain will reach to the VAP-binding domain, and the required length is about 18 carbons (Fig 7B–7E). An unsaturation in the N-linked acyl chain is not required for the chain to kink inside the hydrophobic cavity. This is evident from the previous crystal structures of GLTP with bound fully saturated glycolipids [8, 12, 44, 57].

We have previously shown that GLTP interacts with VAP-A through its FFAT-like motif [58]. We now found that the length of the ceramide backbone N-linked acyl chain clearly influenced how GLTP senses these lipids, in terms of its binding and transfer capacity. Short chains are bound strongly and are transferred rapidly, whereas longer ceramides are bound more weakly and are transferred more slowly. We speculate that the binding of different GlcCers by GLTP can affect the ability of GLTP to interact with VAP.

The VAP proteins exist in hetero- or homo-dimer form, and bring together other components that bind VAP to form complexes [21–24]. We therefore searched for proteins related to sphingolipid synthesis that have FFAT-like motifs and that could be candidates for binding to VAP together with GLTP. We used Levine’s formula [38] to identify FFAT-domains and we targeted proteins involved in the sphingolipid synthesis machinery. We found several putative FFAT-domains (**Table 1**) that could possibly associate with GLTP through VAP-dimer-binding. These proteins that contain putative FFAT-domains include proteins in the fatty acyl-chain synthesis chain, as well as ceramide synthases. Several of these are trans-membrane proteins, and most are known to exist in the ER. In the ceramide synthesis pathway, serine palmitoyltransferase (SPT), ceramide synthases CerS1 and CerS4 had a FFAT-like domain, whereas KSR-reductase (3-ketosphinganine reductase) and DES1 and DES2 (dihydroceramide desaturases) were not highly ranked among the proteins with putative FFAT-like domains.

**Table 1:** List of putative FFAT-like domains found in proteins related to the sphingolipid synthesis.

Protein	Function	Putative FFAT domain	Levine factor
OSBP	Oxysterol-binding protein 1, original FFAT (EFFDAxE) motif	^351^DEDDEN**EFFDA**P**E**II	0
CERT	Ceramide transport protein	^315^SLINEE**EFFDA**V**E**AA	1.0
GLTP	In vitro glycolipid transfer protein	^26^AVSHLP**PFFDC**L**G**SP	3.5
FAPP2	In vivo GlcCer transfer protein	^22^LCGGIL**SYYDS**P**E**DA	3.0
CerS1	C18 ceramide synthase	^316^ELKDLR**EYDTA**E**A**QS	4.0
CerS4	C18 & C20 ceramide synthase	^282^TQILYT**TYYES**I**S**NR	3.5
SMSr	Ceramide & phosphoethanolamine synthesis sensor	^380^IWFPMF**SFFEC**N**V**NG	3.0
SMS1	Sphingomyelin synthase	^23^LENAMP**EYCEP**L**E**HF	4.5
SREBF2	Cholesterol synthesis regulator	^827^QEEESC**EFSSA**L**E**YL	2.0

We used the algorithm by Murphy and Levine [38] to calculate the strength of the FFAT-like motifs.

## Discussion

In this study, we proposed that GLTP can distinguish between short glucosylceramides with N-linked acyl chains of C16 and shorter from those with longer acyl chains. We analyzed changes in the GLTP thermal denaturation using a CPM probe. The melting temperature of wildtype GLTP is 75°C when no GlcCer is bound and lower for all GlcCers bound to GLTP, regardless of the chain length. The CPM approach does not report to any greater significance differences in the melting temperature of the GlcCer-GLTP complex. However, it is clear that a bound GlcCer affects the GLTP melting temperate. We interpret the lower melting temperature of GLTP with bound GlcCers to indicate a higher solvent content in the glycolipid-binding cavity, compared to the empty apo-GLTP. The collapsed hydrophobic tunnel allows for the lowest solvent accessibility and consequently has the highest melting temperature.

We continued with a systematic SPR analysis of the GLTP transfer rate for different GlcCers from POPC-vesicles. We showed that the transfer rates are higher for the shorter acyl-chain GlcCers than for their longer-chain counterparts. We then used a competition transfer assay to analyze how different acyl-chain length GlcCers would change the transfer rate of a fluorescent GlcCer probe. The data showed that longer acyl-chain GlcCers have a lesser effect on the transfer rate when compared to shorter acyl-chain GlcCer. This means that shorter acyl-chain lipids are more easily transported by GLTP, in contrast to the longer acyl-chain GlcCers. The transfer rate of C12:0-GlcCer deviates slightly in the competition assay from the other short GlcCers. This could be due to an interaction between the C11-TopFluor-GlcCer and the C12:0-GlcCer. The effective length including the fluorophore of C11-TopFluor-GlcCer is closest to the C12:0-GlcCer of the short GlcCers.

Since GLTP is intrinsically fluorescent, we used its Trp emission as a measurement of how well C8:0-GlcCer to C24:0-GlcCer would influence the emission blueshift. Addition of phospholipid vesicles alone did not shift the emission of Trp, whereas increasing amounts of added GlcCer resulted in different emission blueshifts that depended on the length of the GlcCer. This again confirmed that C8:0-, C12:0-, and C16:0-GlcCer were able to shift the Trp emission to a significantly greater extent than could the longer C18:0-, C20:0, and C24:0-GlcCer. Taken together, our experiments clearly show that GLTP prefers GlcCers with shorter acyl-chain lengths over longer when it comes to binding and lipid transfer. The direct mechanism for GLTP transfer is not known and most likely involves many steps [59]. First GLTP needs to locate and bind its glycosphingolipid substrate in the membrane outer leaflet, then the GLTP-glycosphingolipid complex desorbs from the membrane interface, diffuses trough the aqueous environment and eventually the GLTP complex binds to another (or the same) membrane leaflet and releases its bound lipid. At least all these steps have an impact on how the GLTP transfer reaction progresses. How strongly the glycosphingolipid is associated with the membrane matrix is going to be affected by the length of the glycosphingolipid and the matrix composition. Therefore, determining the impact of the length of the acyl chains of glycosphingolipids in the transfer process is going to be one of the most important factors in understanding the GLTP mechanism.

We then analyzed the effect of different acyl-chain length glycolipids on GLTP’s ability to bind VAP-A using a GST pull-down experiment. We showed that the binding of GlcCer leads to a lowering of GLTP/VAP-A binding. This indicates a regulating function of glycolipid uptake on GLTP’s association to VAP-A.

The VAP-binding FFAT-domain is on the outside as well as in the inner hydrophobic chamber of the GLTP structure. Therefore, the binding of substrate lipids with different lengths of N-linked acyl chains will likely change the structural conformation of the FFAT-domain and its ability to interact with the VAP protein. Of particular note is the acyl chain kink area, which is just next to the FFAT-domain and is always filled by the bound lipid, regardless whether the lipid is bound with both or only one chain inside the GLTP (sphingosine-in versus sphingosine-out sphingolipid conformation in the crystal structures). A change in the conformation of the FFAT-domain could be the key that induces or hinders GLTP binding to VAP on the ER.

We have analyzed several crystal structures of GLTP, with different bound substrates that vary in sugar groups as well as acyl-chain lengths. In these structures, GLTP binds its lipid substrate in two different ways: in a sphingosine-in or sphingosine-out conformation, with both filling the inner chamber in different ways but still conforming to certain structural restrictions. Based on the crystal structures, both the short and the long N-linked chains appear to bend into the kink area when their sphingosine chain is in an out-conformation. By contrast, when the sphingosine is in the hydrophobic tunnel, the N-linked acyl chain is not bent to the same extent (Fig 7). An interesting and still unanswered question, which could have an impact on the function of GLTP after lipid-uptake, is whether different acyl-chain length glycosphingolipids bind in either the sphingosine-in- or -out conformations depending on their length. The sphingosine chain in the sphingosine-out conformation could perhaps even interact with other GLTPs or with a lipid membrane. However, the GLTP homodimerization has only been seen in crystal structures [57].

If GLTP binding through VAP to the ER is controlled by the binding of its substrate, then GLTP could function as a sensor of the GlcCer levels or, more specifically, of the levels of GlcCers with specific acyl-chain lengths. The ceramide synthase protein family (CerS) creates ceramide by adding acyl chains of different lengths to sphingosine, and this synthesis takes place in the ER [10]. From the ER, ceramide is transported either through vesicular transport mechanisms to downstream glycosphingolipid synthesis or via CERT for sphingomyelin synthesis [60]. The GLTP acyl chain specificity could therefore be tied to a regulation of one or several CerS-family members, perhaps in the form of a feedback loop from the glycosphingolipid branching in the sphingolipid synthesis pathway. This could determine whether GlcCer would remain as GlcCer or whether it would be further processed into LacCer and more complex glycosphingolipids. We have indirect support for this supposition from our earlier work showing that Gb_3_ is downregulated when GLTP is downregulated, and that both Gb3 and GlcCer are upregulated by a GLTP upregulation [61, 62]. Both GLTP up- and downregulation perturb and lower the C16 and C24 acyl-chain glycosphingolipid-levels, while the levels of the majority of the remaining acyl-chain length glycosphingolipids remain unchanged. We have also shown that GLTP-expression levels react only to newly synthesized GlcCer [62, 63].

Our use of Levine’s formula [38] for predicting possible FFAT-domains identified at least 2 CerS—CerS1 and CerS4—that have possible FFAT-domains. We have also identified domains in several other proteins involved in all stages of the sphingolipid synthesis machinery. The VAPs exist in hetero- or homo-dimeric forms [64] and could bring together different components of the ceramide synthesis machinery. Speculatively, at the ER, GLTP could interact with the other putative VAP-binding proteins, including CerS and other proteins needed for the ceramide synthesis complex, thereby adjusting the synthesis of their end-products. The selectivity and sensitivity of this sensor function would most probably be tissue dependent, as the various CerS family members show differing expression levels in different tissues [65–71]. Therefore, GLTP could be more sensitive in this sensor role in select tissues because of the acyl-chain length differences in the GlcCers produced there. We therefore propose a sensor role for GLTP, whereby it can sense different acyl-chain length GlcCers at different levels, depending on the variance in the CerS expressed in a given tissue. GLTP could thereby affect the synthesis of ceramide and thus glycosphingolipids in a feedback loop.

## Supporting information

S1 Fig Typical SPR response curves. After vesicle binding and NaOH wash the vesicles are stable with no loss of material from the chip surface. After GLTP addition, GlcCer is removed and the mass of the bound vesicles decrease and the response in the SPR signal is registered as a decrease in the response units.
(TIFF)

S2 Fig Transfer of C18:0-GlcCer by wild-type GLTP (solid red line) and a transfer inactive GLTP mutant (W96A) from POPC vesicles (dashed red line). The black line shows the control transfer of POPC without GlcCer and the grey line the W96A GLTP mutant. The graph shows representative curves from at least three different experiments.
(TIFF)

S3 Fig Rates of the GLTP mediated GlcCer transfer measured by SPR. (**A)** Average SPR curves of the GLTP transfer of GlcCer. (**B**) The slopes (m) were calculated from the average SPR curves, using linear regression (y = mx + b), and shown by the dashed lines.
(TIFF)

S4 Fig Changes in tryptophan emission of GLTP by different GlcCers. The change in Trp emission for increasing amounts of different fully saturated N-linked acyl chain lengths of GlcCer to GLTP in PBS. The Trp emission scans correspond to GlcCer concentrations of 0, 1.28, 2.55, 3.82, 5.1, 6.38, 7.6, 8.9, 10.2, 11.4 and 12.8 μM with respect to emission intensity. The curves shown are representatives from a series of at least three different experiments.
(TIFF)
