# Supplementary figures and images for: Glucosylceramide acyl chain length is sensed by the glycolipid transfer protein
Source: PLoS One. 2018 Dec 14;13(12):e0209230. doi: 10.1371/journal.pone.0209230 (PMC6294359; doi:10.1371/journal.pone.0209230)

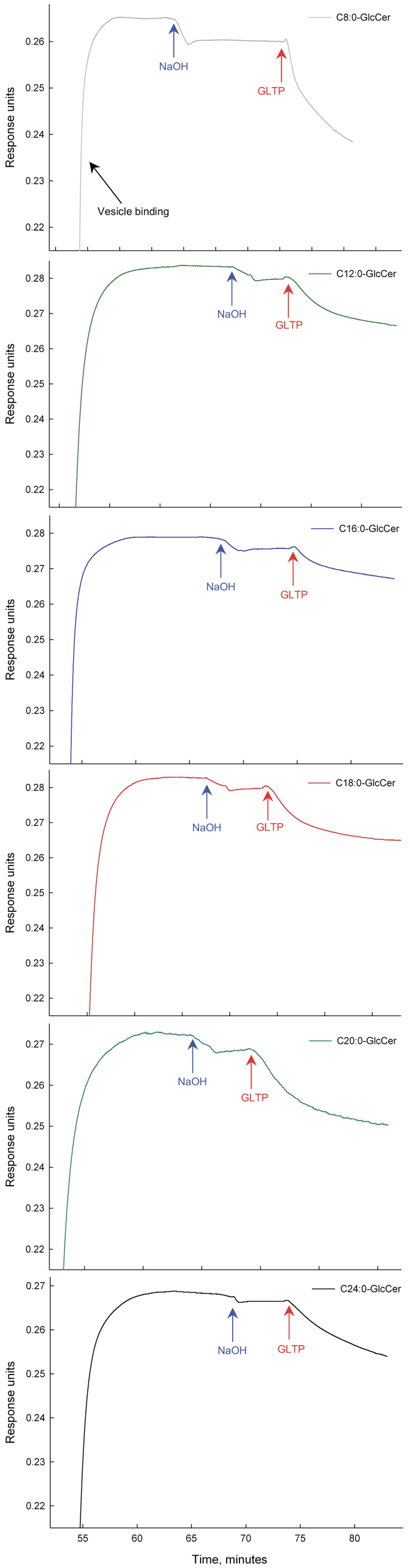

Supplement: S1 Fig — After vesicle binding and NaOH wash the vesicles are stable with no loss of material from the chip surface. After GLTP addition, GlcCer is removed and the mass of the bound vesicles decrease and the response in the SPR signal is registered as a decrease in the response units. (TIFF) [file pone.0209230.s001.tiff]

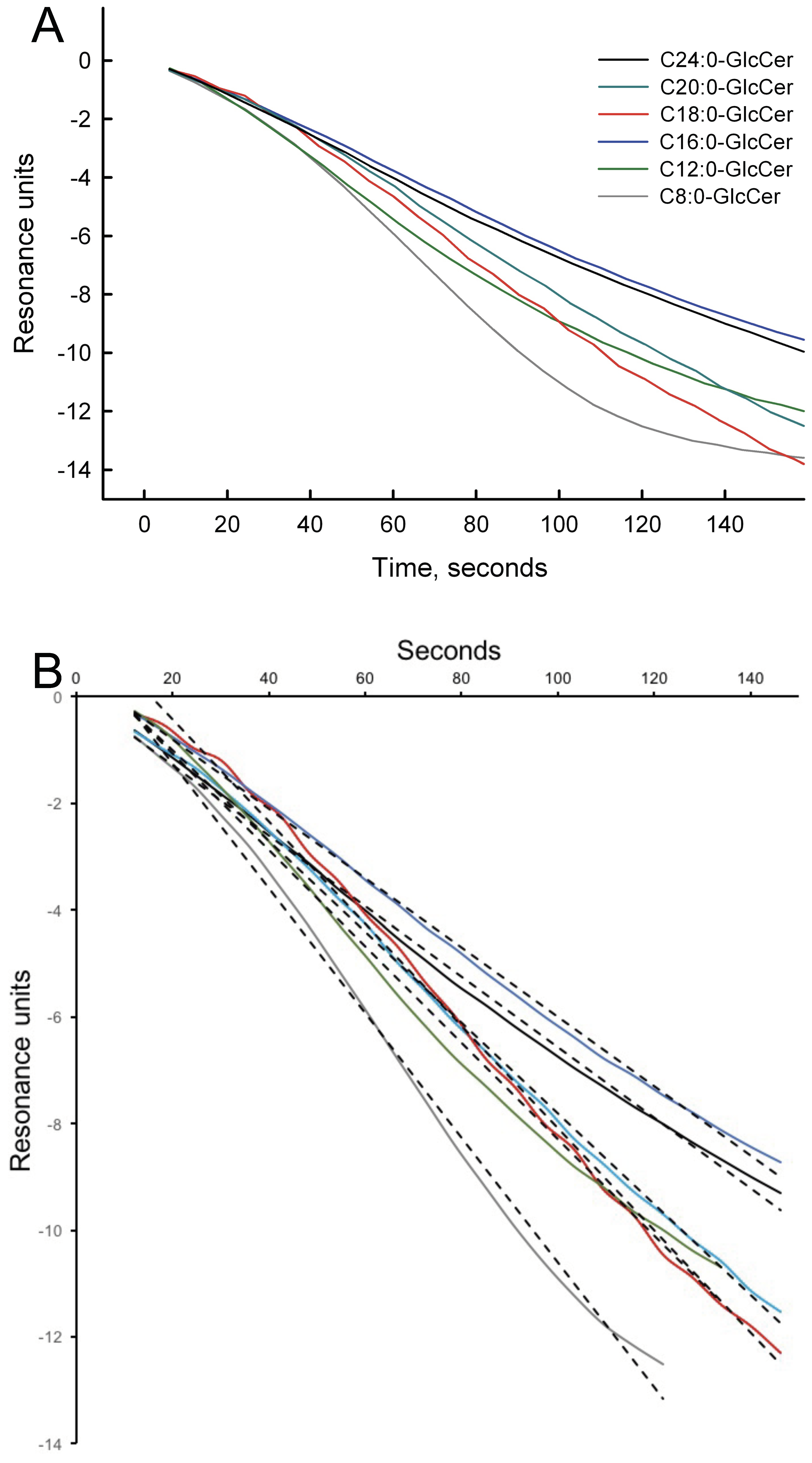

Supplement: S3 Fig — (A) Average SPR curves of the GLTP transfer of GlcCer. (B) The slopes (m) were calculated from the average SPR curves, using linear regression (y = mx + b), and shown by the dashed lines. (TIFF) [file pone.0209230.s003.tiff]

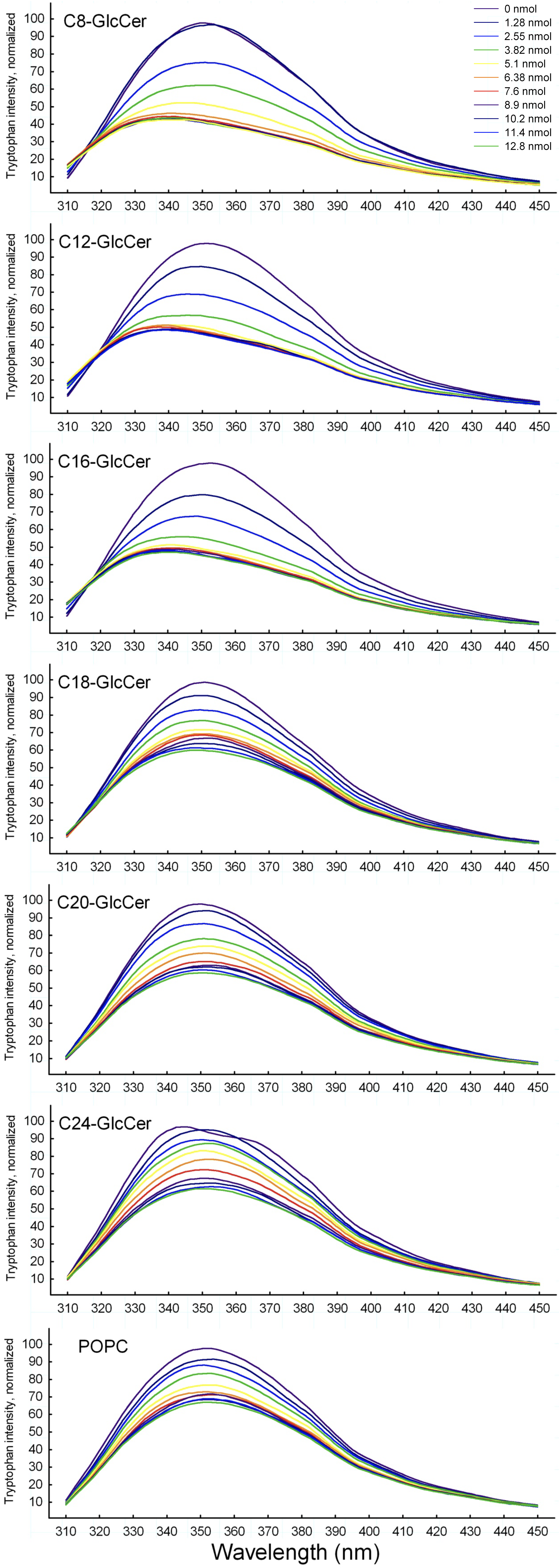

Supplement: S4 Fig — The change in Trp emission for increasing amounts of different fully saturated N-linked acyl chain lengths of GlcCer to GLTP in PBS. The Trp emission scans correspond to GlcCer concentrations of 0, 1.28, 2.55, 3.82, 5.1, 6.38, 7.6, 8.9, 10.2, 11.4 and 12.8 μM with respect to emission intensity. The curves shown are representatives from a series of at least three different experiments. (TIFF) [file pone.0209230.s004.tiff]
